# Experimental Assessment of Different Receiver Structures for Underwater Acoustic Communications over Multipath Channels

**DOI:** 10.3390/s120202118

**Published:** 2012-02-14

**Authors:** Guosong Zhang, Jens M. Hovem, Hefeng Dong

**Affiliations:** Department of Electronics and Telecommunications, Norwegian University of Science and Technology (NTNU), Trondheim NO-7491, Norway; E-Mails: Jens.Hovem@iet.ntnu.no (J.M.H.); Hefeng.Dong@iet.ntnu.no (H.D.)

**Keywords:** underwater acoustic communication, time reversal, passive-phase conjugation, matching pursuit, decision feedback equalizer

## Abstract

Underwater communication channels are often complicated, and in particular multipath propagation may cause intersymbol interference (ISI). This paper addresses how to remove ISI, and evaluates the performance of three different receiver structures and their implementations. Using real data collected in a high-frequency (10–14 kHz) field experiment, the receiver structures are evaluated by off-line data processing. The three structures are multichannel decision feedback equalizer (DFE), passive time reversal receiver (passive-phase conjugation (PPC) with a single channel DFE), and the joint PPC with multichannel DFE. In sparse channels, dominant arrivals represent the channel information, and the matching pursuit (MP) algorithm which exploits the channel sparseness has been investigated for PPC processing. In the assessment, it is found that: (1) it is advantageous to obtain spatial gain using the adaptive multichannel combining scheme; and (2) the MP algorithm improves the performance of communications using PPC processing.

## 1. Introduction

Coherent underwater acoustic communications are challenged by acoustic channels, which are often characterized as time-varying, dispersive, sparse, *etc*. [1]. Therefore, much of the recent research has been focused on the development of channel equalizers to remove intersymbol interference (ISI) in multipath environments, especially for high-rate coherent communications.

One receiver cannot avoid deep fading in time-varying channels, and thus the equalizers fail to remove ISI. With multiple sensors exploiting spatial diversity, Stojanovic *et al.* [2] has proposed a multichannel decision feedback equalizer (McDFE). The disadvantage of McDFE is its complexity due to the computational load, which increases with the time spread of underwater channels. Therefore, it is difficult to apply McDFE in underwater channels of long time spread, especially using a large number of receiving hydrophones.

Another novel method is the time reversal mirror (TRM), originally proposed by Fink [3], which was later applied for underwater communications. The focusing of TRM results in a significant reduction of ISI for underwater communications, which has been demonstrated by Edelman *et al.* [4,5]. Two vertical hydrophone arrays and two-way transmission are required by TRM to achieve the focusing at the transmitter. During the transmission, the underwater channel is required to be constant.

An alternative technique for underwater communications is proposed by Rouseff *et al.* [6] to take advantage of the focusing at the receiver, commonly referred as passive time reversal or passive-phase conjugation (PPC). This method requires only one receiving array and one-way transmission. ISI cannot be eliminated by the focusing, and thus a subsequent channel equalizer is used to remove residual ISI [6–9], where a single channel decision feedback equalizer (DFE) is used. It is referred to as PPC-DFE in this paper. Spatial diversity is used by the focusing to suppress ISI. In a real oceanic environment, it is difficult to predict time variant spatial coherence [10], when interchannel correlations impact spatial focusing.

Stojanovic [11] has discussed the upper bound performance of time reversal communications, but it is very difficult to predict real performance of time reversal communications, as spatial coherence is neglected in the model. By numerical simulations and experimental demonstrations, Yang [12] has demonstrated that McDFE achieves superior performance over that of PPC-DFE. This leads to a receiver structure which uses adaptive multichannel combining after PPC processing in each individual channel.

Zhang *et al.* [13] have presented a receiver structure—joint PPC and McDFE (PPC-McDFE). This receiver structure involves temporal focusing (pulse compression by PPC processing) for time delayed arrivals [14], and thus the computational load of a subsequent McDFE is much reduced. It is well known that temporal focusing degrades with time evolution in time-varying channels. To counter for this degradation, the block-based approach proposed by Song [15] can be used to extend PPC-McDFE in time-varying channels.

PPC processing requires information of the channel characteristics, which can be estimated using training symbols. Underwater channels are often sparse, especially at the high-frequency regime, where there are a few dominant arrivals. The dominant arrivals can be estimated using the matching pursuit (MP) algorithm [16]. Song [17] has shown that the MP algorithm exploits the channel sparseness to improve the performance of PPC-DFE. It is an open question whether the MP algorithm can improve the performance of PPC-McDFE, in comparison with the conventional channel estimation method—the least squares (LS) method.

The above brief introduction shows that different approaches have been proposed and have been tested in field experiments. However, the experiments were conducted under different conditions, and it is therefore difficult to compare the performance of different receiver structures. This has motivated the work of this paper. A recent field experiment was conducted to collect data over a range of 7.4 km, when three modulation schemes were used. Four data rates with a maximal data rate of 4 kilo-bits/s have been achieved. Using the same real data, we compare the performance of three receiver structures: McDFE, PPC-DFE, and PPC-McDFE. These structures are frequently discussed in the literature, and in the future we may extend the discussion to other structures and modulation schemes.

As required, information of the channel characteristics for PPC processing can be obtained by a channel probe signal or estimated using training symbols. For example, using a linear frequency modulation pulse (LFM) chirp as a channel probe signal, when the chirp is also used as a shaping pulse at the transmitter, the received LFM is immediately used for PPC processing. Alternatively, the channel is estimated using training symbols, when a root-raised-cosine pulse (RRC) is used as a shaping pulse. In this paper, we have also tested the scenario using the two shaping pulses.

The contributions of this paper include: (1) experimental assessment of the difference between two shaping pulses—LFM and RRC; (2) performance comparison of the McDFE, PPC-DFE, and PPC-McDFE structures; (3) evaluation of the block-based approach for PPC-McDFE; and (4) assessment of the MP algorithm for both PPC-DFE and PPC-McDFE, in which PPC processing is implemented in two modes—one block and multi-block.

This paper is organized as follows: Section 2 introduces the field experiment conducted in Trondheim harbor on 7 September 2011. Section 3 shows the receiver structures: (1) McDFE; (2) PPC-DFE; and (3) PPC-McDFE. Section 4 briefly introduces channel estimations for PPC processing, the LS method and the MP algorithm. In Section 5, the results are presented and discussed, and performance of the three structures is shown. Finally, Section 6 summarizes the work.

## 2. The Experiment

### 2.1. The Setup

The communication experiment was conducted on 7 September 2011, in Trondheim harbor (Norway), where the water depth varies from 10 m to 400 m. The transmitter was carried by the NTNU research vessel R/V Gunnerus, and it used a hemispherical acoustic transducer deployed at a depth of 20 m. The dynamic positioning system of R/V Gunnerus was activated during the trial to reduce drifting.

A cross receiving array of 12 hydrophones was deployed from a pier, where the water depth was about 10 m. The array consisted of a vertical array of eight hydrophones (hydrophones No. 1–8) with 1 m element spacing and a horizontal array with four hydrophones (hydrophones No. 9–12) with 1.5 m element spacing. Hydrophone No. 1 was located 0.5 m below the sea surface, and the depth of the horizontal array was 4.5 m. The range between the source and the receiving array was 7.4 km.

Digital modulations of phase shift keying (BPSK), quadrature phase shift keying (QPSK), and eight quadrature amplitude modulation (8QAM) were used. The carrier frequency of the transmitted signal was 12 kHz. A 0.1 s LFM chirp with a Hanning window was used for coarse time synchronization in each data packet, and its effective bandwidth was 2.2 kHz. When the LFM was used as the channel probe signal, it was also used as a shaping pulse. As a shaping pulse, the roll-off coefficient of RCC was 1.

**Figure 1.**
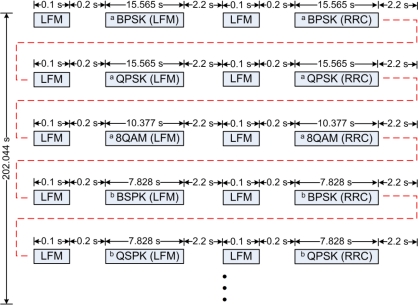
Block diagrams of the transmitted signals using different shaping pulses shown in the parentheses. ^a^ The symbol rate was 1 kilo-symbol/s; ^b^ The symbols rate was 2 kilo-symbol/s.

Figure 1 shows the signals which were repeatedly transmitted every 202.044 s for 15 periods. The signals of the same modulations, but using different shaping pulses were transmitted continuously with a time gap of 2.2 s. The symbol rates were 1 kilo-symbols/s and 2 kilo-symbols/s, and the respective bandwidths were 2 and 4 kHz when the RRC was used. The received waveforms were recorded at a sampling frequency of 96 kHz for offline processing in the laboratory.

### 2.2. Channel Characterization and Measurements

**Figure 2.**
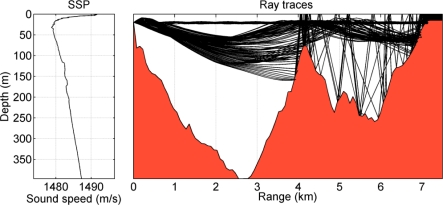
Measured SSP (the left panel) and the ray traces (the right panel) from a source on the left. The source was at a depth of 20 m.

Sound speed profile (SSP) measured by the R/V Gunnerus is shown by the left panel of Figure 2. The sound speed profile has a surface channel and a negative gradient down to about 50 m. At deeper depths, the sound speed increases nearly linearly. With the conditions of the SPP and the bathymetry, the PlaneRay ray-tracing program [18] is used to illustrate the acoustic propagation during the trial. The right panel of Figure 2 shows that ray traces and the bathymetry from a source at 20 m depth to the receiving array located at a distance of 7.4 km. The sound propagation dominated by the sound channel at about 25 m and the positive gradient below 50 m. It is shown that there are several almost horizontal paths in the sound channel as well as several deep refracted paths, and all other possible ray paths are blocked by the seamount at 4 km.

**Figure 3.**
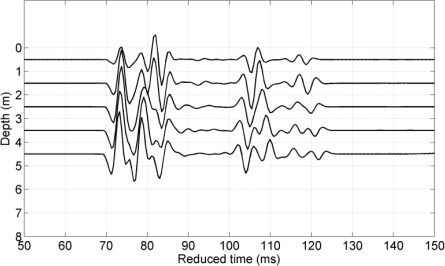
Modeled channel impulse response calculated by the PlaneRay program to the vertical array with five hydrophones spanning the depth of 0.5 m to 4.5 m.

Figure 3 shows the simulated responses to the vertical array with five hydrophones spanning the depth of 0.5 m to 4.5 m. The results are plotted as a function of reduced time, which is the actual travel time with the nominal gross travel time of 4.9333 seconds subtracted. The transmitted pulse used in the simulations was a short transient with 2 ms duration. There is a group of arrivals followed by a second group arriving about 40 ms later. This structure can be understood from the ray tracing with the first group is due to the sound channel paths and the second is the deep refracted paths. Each of the groups has several multipath contributions probably caused by a multitude of surface and bottom reflections occurring in shallow area near the receiving array.

**Figure 4.**
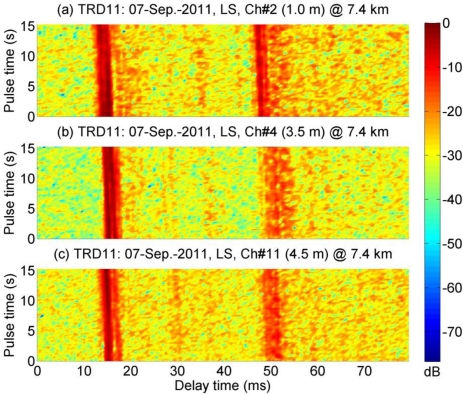
Channel response at different depths. (**a**) 1 m; (**b**) 3.5 m; (**c**) 4.5 m.

Figure 4 shows examples of channel response estimated using the LS method. Within the observations of 15 s, the responses varied with time. In each receiving channel, there are two groups of concentrated arrivals with a time span of 35 ms, and they correlate with the simulated results in Figure 3. It is evident that the channel is sparse. It is apparent that they are similar over the three water depths. In particular, the signals received in Ch #2 and Ch #4 are highly correlated, the correlation coefficient between these two channels is calculated to be 0.68.

## 3. The Receiver Structures

Generally, the receiver recovers distorted information by baseband signal processing, where multipath channels are often modeled as finite filters of multiple taps. In digitized form, the received signal at *k*th hydrophone can be written as:

(1) $$V_{k}^{n}=\sum_{l=0}^{L-1}H_{k}^{l}I_{n-l}+W_{k}^{n},\;k=1,\ldots ,K$$

where $H_{k}^{l}$ denotes the *l*th tap of channel impulse response (CIR) *H_k_* which spans *L* symbol interval, *I_n_* is the *n*th symbol of a sequence {*I_n_*}, and $W_{k}^{n}$ represents a bandwidth limited noise. In a multipath channel, where *L* ≠ 1, ISI caused by *H_k_* results in errors. The objective of a channel equalizer is to remove the ISI.

**Figure 5.**
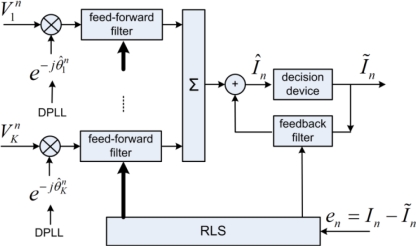
Block diagram of McDFE using the RLS algorithm. There are *K* receiving channels. ${\hat{\theta }}_{k}^{n}$ denotes the estimate of phase offset $\theta_{k}^{n}$ at the *k*th receiving channel, *Î_n_* presents the soft estimate of *I_n_*, and *Ĩ_n_* is the decided symbol which best matches *Î_n_*.

Figure 5 shows the block diagram of McDFE [2]. The tap coefficients of the *K*-channel feed-forward filters plus one channel feedback filter are jointly updated by the recursive least squares (RLS) algorithm for its fast rate of convergence [19]. The technique of a second order digital phase-locked loop (DPLL) is implemented for the carrier-phase tracking. The DPLLs $\left\{e^{-j{\hat{\theta }}_{k}^{n}}\right\}$ operate on a symbol-by-symbol basis to remove phase changes caused by the carrier frequency shift. In order to deconvolve *H_k_*, the number of taps for the McDFE is determined by the time spread *L*, and it is usually chosen in an *ad hoc* manner. The computational load increases with *L*, and it may become prohibitive, when a large number of hydrophones are used. Moreover, under the same channel conditions, the number of taps increases with the symbol rate.

**Figure 6.**
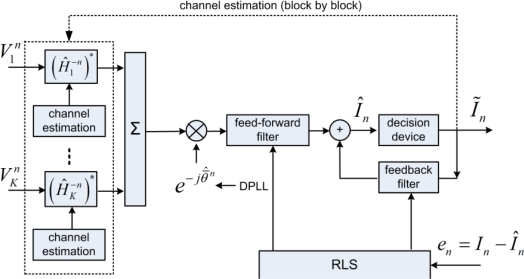
Block diagram of passive time reversal receiver structure PPC-DFE. ${\left({\hat{H}}_{k}^{-n}\right)}^{*}$ denotes complex conjugation of the time reversed channel estimate ${\hat{H}}_{k}^{n}$. ${\hat{\bar{\theta }}}_{k}^{n}$ denotes the estimate of the phase offset ${\bar{\theta }}_{k}^{n}$ after focusing.

Figure 6 shows the receiver structure of passive time reversal—PPC-DFE. Following the focusing, only one channel DFE is required to remove residual ISI [8], when one DPLL is implemented for carrier-phase tracking. The focusing mitigates ISI, the number of taps for the one channel DFE is much reduced, and thus the complexity of PPC-DFE is much lower than that of McDFE. Note that the focusing degrades with time in time-varying channels.

As suggested by Song [15], a block-based approach extends PPC-DFE to be implemented in time-varying channels. The idea is that channel estimations $\left\{{\hat{H}}_{k}^{n}\right\}$ are updated on a block-by-block basis, when the channel is assumed time-invariant within each block of a short time interval. The channel estimations $\left\{{\hat{H}}_{k}^{n}\right\}$ are subsequently updated using detected symbols in the previous block. This block-based approach does not change the basic principle of TR focusing, which obtains spatial diversity by to mitigate ISI. Zhang *et al*. [13] has discussed the impact of the time variant interchannel correlations on the performance of PPC-DFE.

**Figure 7.**
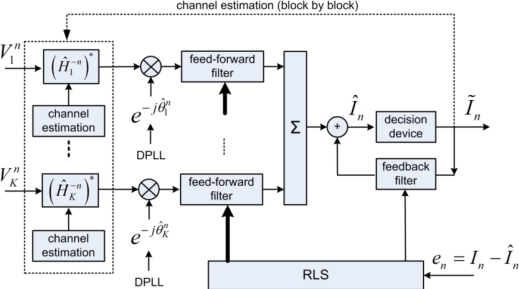
Block diagram of PPC-McDFE.

The receiver structure PPC-McDFE is shown of Figure 7. Here, pulse compression is achieved by PPC processing in each individual channel, and then a subsequent McDFE is implemented to remove residual ISI by adaptive multichannel combining. The RLS algorithm updates the tap coefficients to minimize output mean square error (MSE). Pulse compression is achieved in the same way for single receiver [20,21]. Thus it is used by PPC-McDFE to reduce the complexity of the subsequent McDFE which obtains spatial gain. As discussed by Yang [7], the peak-to-sidelobe ratio of pulse compression is determined by the channel response, while the pulse compression acts as a rake receiver recombining time delayed arrivals. In time-varying channels, PPC-McDFE can be extended using the block-based approach [15].

## 4. Channel Estimations

This section briefly introduces two channel estimation methods. Using training symbols, the channel estimations for PPC processing can be obtained using both the LS method [22] and the MP algorithm [16].

By combining M observed symbols, Equation (1) is rewritten as:

(2) $$\left[\begin{array}{l} V_{k}^{n-M+1}\\ V_{k}^{n-M}\\ \vdots \\ V_{k}^{n} \end{array}\right]=\left[\begin{array}{lll} I_{n-M+1} & \cdots & I_{n-M-L+2}\\ \vdots & & \vdots \\ I_{n} & \ldots & I_{n-L+1} \end{array}\right]\;\left[\begin{array}{l} H_{k}^{0}\\ H_{k}^{1}\\ \vdots \\ H_{k}^{L-1} \end{array}\right]+\left[\begin{array}{l} W_{k}^{n-M+1}\\ W_{k}^{n-M}\\ \vdots \\ W_{k}^{n} \end{array}\right]$$

which is simplified to:

(3) $$V_{k}={\mathrm{IH}}_{k}+W_{k}$$

In the channel estimation problem, the information matrix *I* is known as training symbols. An estimation of *Ĥ_k_* can be obtained by solving LS problem:

(4) $${\hat{H}}_{k}=\text{arg}\;\underset{{\tilde{H}}_{k}}{\text{min}}\left\{{\left\|V_{k}-I{\tilde{H}}_{k}\right\|}^{2}\right\}$$

which gives the solution:

(5) $${\hat{H}}_{k}={\left[I^{H}\;I\right]}^{-1}I^{H}\;V_{k}$$

In practice, the LS method is sensitive to noise. When a channel is sparse, the CIR consists of a large number of zeros among several dominant taps, and the LS method will suffer from the noise between dominant taps. Besides, the LS method involves matrix inversion, and it sometimes suffers ill-conditioned problem of a matrix of large eigenvalue spread.

To exploit the sparse property of channels, the channel estimation problem can be reconsidered as an approximation problem. It is assumed that the received signal vector is approximated by:

(6) $${\left({\hat{V}}_{k}\right)}_{M}=\sum_{i=0}^{M-1}{\hat{H}}_{k}^{p_{i}}{\left(I\right)}_{p_{i}}$$

where (*I*)_*p*_*i*__ is the *p_i_* th column of information matrix *I*. Finding the approximation of (*V̂_k_*)*_M_* that minimizes ||(*V̂_k_*)*_M_* – *V_k_*|| is an non-deterministic polynomial-time hard problem [23,24], which means there is unknown polynomial time algorithm that can solve this problem. MP [16] is a greedy algorithm that achieves non-optimal yet computational efficient approximation of *V_k_*.

The MP algorithm selects one column in matrix *I* which is best aligned with residual signal *r*_*p*−1_, where *r*_0_ = *V_k_* at initial step. In practice at the *p*th step, the selected *l_p_* th column of *I* is determined by:

(7) $$l_{p}=\underset{l}{\text{arg}\;\text{max}}\left\{\left\|{\left(I\right)}_{l}^{H}r_{p-1}\right\|/\left\|{\left(I\right)}_{l}\right\|\right\}$$

Correspondingly, the tap value ${\hat{H}}_{k}^{l_{p}}$ is estimated by:

(8) $${\hat{H}}_{k}^{l_{p}}=\frac{{\left(I\right)}_{l_{p}}^{H}\;r_{p-1}}{{\left\|{\left(I\right)}_{l_{p}}\right\|}^{2}}$$

and *r_p_* is updated by:

(9) $$r_{p}=r_{p-1}-\frac{{\left(I\right)}_{l_{p}}^{H}\;r_{p-1}}{{\left\|{\left(I\right)}_{l_{p}}\right\|}^{2}}{\left(I\right)}_{l_{p}}$$

This iteration is terminated until the preset *P* taps have been estimated. In practice, one column in *I* is probably selected more than once. To deal with this problem, we can either exclude previously selected columns in the search press shown in Equation (7), or the tap value calculated in Equation (8) can be added to the value found in previous steps [25]. In this paper, we use the former method.

## 5. Results and Analysis

Recorded signals of 15 periods are processed with parameters given in Table 1, in which some are chosen in an *ad hoc* manner. For instance, the number of taps *N*^1^*_ff_*, and *N*^1^*_fb_*. As suggested by Stojanovic [26], the integral tracking constant *K*_2_ is chosen as 10 time smaller than the proportional tracking constant *K*_1_. In subsections of 5.2 and 5.3, the performance of McDFE is selected as a benchmark.

### 5.1. Performance Using Different Shaping Pulses

As mentioned in Section 2.1, both LFM and RRC were used as shaping pulses. In the scenario of using LFM as a shaping pulse, the peak-to-average power ratio (PAPR) is large [9], and it could result in lower power efficiency for a linear amplifier. With a constant transmission power, the source level is reduced. However, the advantage is that the received channel probe signal is immediately used for PPC processing. Using RRC as a shaping pulse, PAPR is reduced, and then the channel response is estimated using different methods.

**Figure 8.**
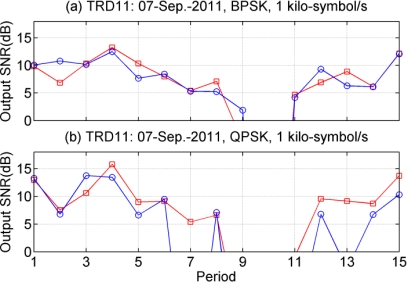
Performance of PPC-DFE using different shaping pulses. (**a**) BPSK; (**b**) QPSK. LFM is used ( 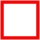), and RRC is used ( 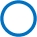).

In terms of output signal-to-noise ratio (SNR), Figure 8 shows the performance of PPC-DFE using different shaping pulses, where the symbol rate is 1 kilo-symbol/s. Due to low input SNRs in the 10th period of Figure 8(a) and the 9th–11th periods of Figure 8(b), the receiver structures fail to recover distorted information. For RRC shaping pulse, the LS method is used to estimate the CIR within 40 ms. The observed time variant performance of PPC-DFE may be caused by the channel variations which resulted in sometimes a low input SNR, as for example at the 10th period. Generally the performance difference between LFM and RRC is small for BPSK, as shown in Figure 8(a). In Figure 8(b), there are small differences over the 9 periods, and large differences in other periods, in particular for 7th and 13th periods. Channel estimations obtained by the LS method are impacted by the noise in the scenarios of low input SNRs, and thus using a LFM as a shaping pulse has shown its advantage. There is also a spreading gain by using the LFM as the shaping pulse, since the bandwidth of the LFM of 2.2 kHz is larger than the signals bandwidth of 1 kHz.

### 5.2. The One-Block Approach

In this subsection, the channel is estimated only once for PPC processing in each data packet. The channel is estimated using training symbols, which are specified symbols in the beginning of communications. Following PPC processing, ISI is removed by the adaptive channel equalizers.

**Figure 9.**
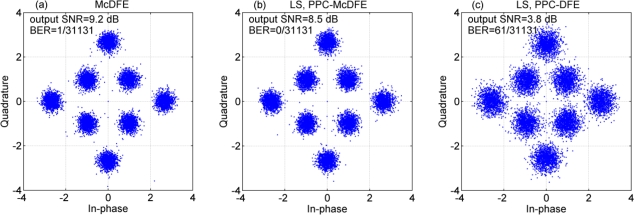
Scatter plot of estimated 8-QAM symbols using different receiver structures. (**a**) McDFE; (**b**) PPC-McDFE; (**c**) PPC-DFE.

Figure 9 shows scatter plots of soft output estimates {*Î_n_*} of different receiver structures, where the LS method is used to estimate the channel for PPC processing. The output SNR given by (1-MSE)/MSE, and the bit error rate (BERs) are given in the legends. Obviously, McDFE achieves the best performance with an output SNR of 9.2 dB, PPC-McDFE approximates the performance of McDFE with an output SNR of 8.5 dB, and PPC-DFE achieves the worst performance with an output SNR of 3.8 dB and a BER of 2.0e–3. As shown in Section 3, the difference between PPC-McDFE and PPC-DFE is the multichannel combining scheme.

**Figure 10.**
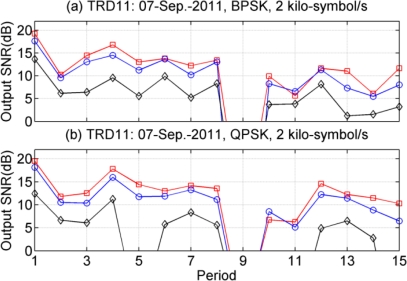
Performance in terms of output SNR for different modulations. (**a**) BPSK; (**b**) QPSK. McDFE ( 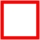), PPC-McDFE ( 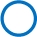), and PPC-DFE (♦).

The results of 15 periods are shown in Figure 10, where the symbol rate is 2 kilo-symbol/s. PPC-DFE achieves the worst performance for both BPSK and QPSK, and obviously it fails in several periods for QPSK. It is apparent that the performance of PPC-McDFE consistently follows that of McDFE. For BPSK shown in Figure 10(a), PPC-McDFE overtakes PPC-DFE with a maximum 6.6 dB output SNR (the 3rd period) and a minimum 2.7 dB output SNR (the 11th period).

**Figure 11.**
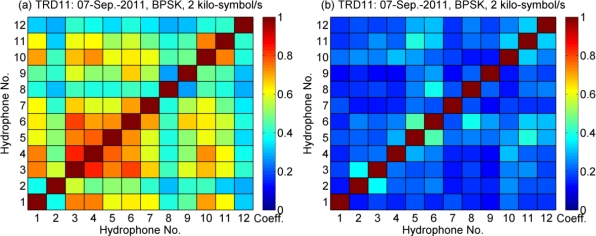
Spatial coherence in different periods. (**a**) The 3rd period; (**b**) The 11th period.

Figure 11 shows the spatial coherence measured in the 3rd and 11th periods, respectively. The spatial coherence between the *k*th and *m*th channel is calculated by:

(10) $$\psi \left(k,m\right)=\frac{{\left|r_{k}\left(-t\right)⊗r_{m}\left(t\right)\right|}_{\text{max}}}{\sqrt{{\left|r_{k}\left(-t\right)⊗r_{k}\left(t\right)\right|}_{\text{max}}{\left|r_{m}\left(-t\right)⊗r_{m}\left(t\right)\right|}_{\text{max}}}}$$

where |*r_k_*(−*t*)⊗*r_m_*(*t*)|_max_ denotes the maximum absolute value of the correlation between *r_k_*(*t*) and *r_m_*(*t*), and *r_k_*(*t*) is the received signal of the *k*th hydrophone. Interchannel correlations shown in Figure 11(a) are stronger than those shown in Figure 11(b), where the time elapse between these two periods is 1,760 s. For instance, the correlation coefficient between Ch #3 and Ch #4 is 0.84 in Figure 11(a), and it is 0.23 in Figure 11(b). Furthermore, in Figure 11(a), it is interesting that there are stronger correlations among the signals received by the vertical array (Ch #1–8) than those among the signals received by the horizontal array (Ch #9–12).

The strength of interchannel correlations correlates with the performance difference between PPC-McDFE and PPC-DFE, which is shown in Figure 10(a). Since it is difficult to predict the time variant spatial coherence in a real oceanic environment [10], it is advantageous to obtain spatial gain using the adaptive multichannel combining, especially in the scenario of a small number of receivers. Thus it is preferable to use McDFE in a channel of short time spread, while PPC-McDFE is suggested in a channel of long time spread.

As shown in Figure 4, the channel impulse response is sparse. This property can be exploited by the MP algorithm. The conventional LS method obtains values for the taps that should be zero in sparse channels, and the MP algorithm only estimates dominant arrivals. For both methods, it is required that the time window is long enough to include all time-spanned arrivals that cause ISI. For the LS method, the time window should not be too long, since a long window may introduce unnecessary noise in the estimate.

**Figure 12.**
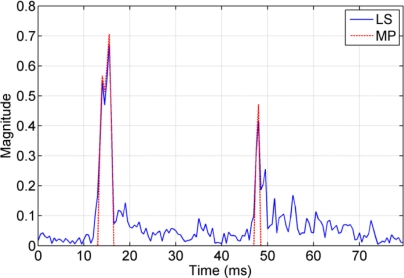
CIR estimated by the LS method and the MP algorithm.

Figure 12 shows the CIR obtained by both LS and MP methods. The number of taps for the MP algorithm was preset to *P* = 4 and finds two main peaks at 12–15 ms, and another main peak at 48 ms. This observation supports the earlier findings there are two main groups of arrivals separated by approximately 35–40 ms. The MP algorithm estimates the same dominant arrivals, but the LS algorithm introduces noise-like values for the taps that should be zeros.

**Figure 13.**
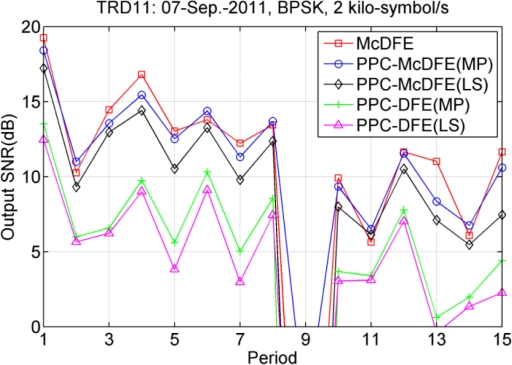
Performance in terms of output SNR at a symbol rate of 2 kilo-symbol/s.

Using both the MP and LS algorithms, performances of PPC-McDFE and PPC-DFE are compared. Figure 13 show the performance comparison at a symbol rate of 2 kilo-symbol/s. The performance of the three structures changes with time, as measured in period. Using the MP algorithm, the performance of both PPC-McDFE and PPC-DFE is improved in most periods. Even though the performance of PPC-DFE is improved by the MP algorithm, e.g., 2.1 dB in maximum (the 15th period), it is still far less than the performance of PPC-McDFE, which overtakes that of PPC-DFE from 3.1 dB (the 11th period) to 7.0 dB (the 3rd period). In average, McDFE leads the performance. In this performance evaluation, it is important to consider the computational time. Based on the same personal computer, McDFE consumed about 20 times computational time than PPC-McDFE (MP) to achieve the approximate performance.

### 5.3. The Multi-Block Approach

It is well known that pulse compression degrades with time evolution, as the channel is time variant in practice. In the Section 5.2, the degradation is neglected, where the subsequent adaptive channel equalizers manage to track the channel variations. In the current subsection, the multi-block approach is used to counter for the variations within each data packet. It is understood that the channel can be assumed constant within a short time interval, correspondingly a data block.

**Figure 14.**
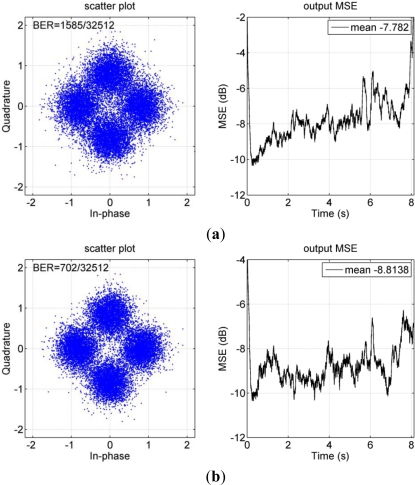
Performance of PPC-DFE with different approaches. (**a**) One block; (**b**) 9 blocks. The MP algorithm is used, and the symbol rate is 2 kilo-symbol/s.

Figure 14 shows an example of performance comparison between one-block and multi-block approaches. For the multi-block approach, the received data packet of 8.128 s was split into eight blocks of 1 s each and one block of 0.128 s. The right panel of Figure 14(a) shows that the single channel DFE encounters difficulties in tracking the channel variations, as the output MSE increases with time. As shown in the left panels of Figure 14, BER is reduced from 4.9% to 2.2%, when the multi-block approach is implemented. As follows for the multi-block approach, each block has time duration of 1 s.

**Figure 15.**
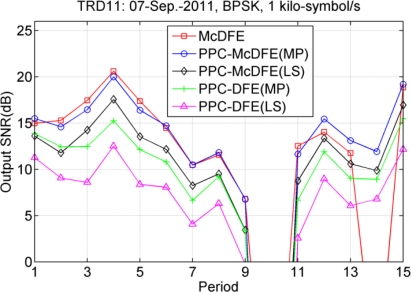
Performance of three receiver structures. There are 16 blocks for PPC processing.

Figure 15 shows the performance assessment, in which the multi-block approach is used for PPC processing. Performance of PPC-McDFE still consistently approximates that of McDFE, and PPC-DFE achieves the worst performance. McDFE fails in the 14th period, which may be due to the impropriate parameters for McDFE, while both PPC-McDFE and PPC-DFE succeed in recovering the distorted information. In observation, the MP algorithm shows advantages over the LS method for the multi-block approach. For instance, using the MP algorithm, 4.1 dB improvement (the 11th period) is obtained by PPC-DFE, and 3.3 dB improvement (the 9th period) is obtained by PPC-McDFE.

The multi-block approach operates on the decision directed mode, and hence there is the issue of error-propagation. In the scenario of low input SNR, the LS method is sensitive to errors of detected symbols of the previous block, while the MP algorithm estimates only dominant arrivals with less impact from the errors. Temporal focusing is more enhanced by the MP algorithm, which leads to better performance. Therefore, the MP algorithm is suggested for the multi-block approach.

**Figure 16.**
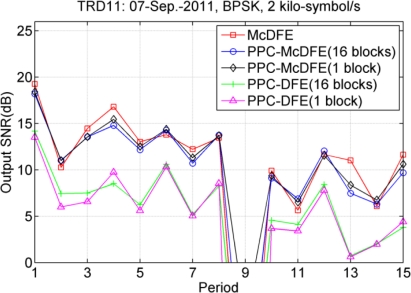
Performance comparison between the one block approach and the multi-block approach.

The multi-block approach may be better than the one block approach, but it depends on the rate of channel variation. Figure 16 shows the comparison between using the one block and multi-block approaches. For PPC-McDFE, there is trivial improvement with a maximum improvement of 0.5 dB (the 12nd period). For PPC-DFE, there is improvement in 11th periods, with mean improvement of 0.6 dB, and the maximum improvement is 1.4 dB (the 2nd period). In the current case, only modest improvement has been obtained when using the multi-block approach, and this can be understood that the collected data were moderately time variant. The multi-block approach cannot avoid the issue of error-propagation, and hence caution should be paid when using this approach, especially in the scenario with low input SNRs.

## 6. Summary and Conclusions

Three receiver structures have been assessed by processing data collected in a recent experiment conducted over a range of 7.4 km. In this high frequency (10–14 kHz) experiment, coherent underwater communications of different symbol rates were achieved, e.g., 1 to 2 kilo-symbol/s. In a large time scale, in terms of period of 202.044 s, the time-variant characteristics of underwater channel are observed by the communication results in terms of output SNR.

As shaping pulses, it is shown that the difference between LFM and RRC is minimal. The LFM shaping pulse provides a simple method for PPC processing, where the received channel probe signal of LFM is immediately used. Using a RRC shaping pulse, it is flexible to select a channel estimation method for PPC processing, e.g., the MP algorithm. In addition, the block-based approach can be implemented in time-varying channels.

As evident, PPC-DFE achieves the worst performance in the assessment, and the performance of PPC-McDFE approximates that of McDFE. Time-variant reverberations result in unpredictable spatial coherence, which may impact on the performance of PPC-DFE. Therefore, it is preferable that the adaptive multichannel combining obtains much spatial gain, especially in the scenarios of a small number of receivers. For instance, it is preferable to use PPC-McDFE instead of PPC-DFE in a channel of long time spread.

In the sparse channel, the MP algorithm has been assessed in two modes. One is the conventional single block approach, and the other is the multi-block approach. The multi-block approach assumes that the channel is constant within each block of a short time interval, and then PPC processing is extended to time-varying channels. Comparing with PPC-DFE, PPC-McDFE is less sensitive to the channel variations. It has been demonstrated that the MP algorithm improves the performance of communications using PPC processing, and thus the MP algorithm is suggested in sparse channels.

## Figures and Tables

**Table 1.** Parameters used in the signal processing of the three receiver structures.

**Parameters**	**Description**	**Value**
*F_s_*	Sampling frequency at the receiver	96 kHz
*f_c_*	Carrier frequency	12 kHz
*R*	Symbol rate	1, 2 kilo-symbol/s
*P*	Number of taps in the MP processing	4
*N*	Over sampling factor	2
*N*^1^*_ff_*	Number of the feed-forward filter taps (McDFE)	20
*N*^1^*_fb_*	Number of the feedback filter taps (McDFE)	5
*N*^2^*_ff_*	Number of the feed-forward filter taps (PPC-DFE)	8
*N*^2^*_fb_*	Number of the feedback filter taps (PPC-DFE)	2
*N*^3^*_ff_*	Number of the feed-forward filter taps (PPC-McDFE)	8
*N*^3^*_fb_*	Number of the feedback filter taps (PPC-McDFE)	2
*T_block_*	Time duration of each block	1 s
*λ*	RLS forgetting factor	0.999
*K*	Number of receiving channels	12
*K*_1_	Proportional tracking constant in DPLL	0.01
*K*_2_	Integral tracking constant in DPLL	0.001
